# Neurotransmitter Genes in the Nucleus Accumbens That Are Involved in the Development of a Behavioral Pathology After Positive Fighting Experiences and Their Deprivation: A Conceptual Paradigm for Data Analysis

**DOI:** 10.3390/ijms26178580

**Published:** 2025-09-03

**Authors:** Natalia N. Kudryavtseva, Dmitry A. Smagin, Olga E. Redina, Irina L. Kovalenko, Anna G. Galyamina, Vladimir N. Babenko

**Affiliations:** 1 Pavlov Institute of Physiology RAS, Saint Petersburg 199034, Russia; 2 FRC Institute of Cytology and Genetics, SB RAS, Novosibirsk 630090, Russia; smagin@bionet.nsc.ru (D.A.S.); oredina@bionet.nsc.ru (O.E.R.); koir@bionet.nsc.ru (I.L.K.); galyamina@bionet.nsc.ru (A.G.G.); bob@bionet.nsc.ru (V.N.B.)

**Keywords:** nucleus accumbens, positive fighting experience, fighting deprivation, neurotransmitter differentially expressed gene

## Abstract

It has been shown previously that repeated positive fighting experience in daily agonistic interactions is accompanied by the development of psychosis-like behavior, with signs of an addiction-like state associated with changes in the expression of genes encoding the proteins involved in the main neurotransmitter events in some brain regions of aggressive male mice. Fighting deprivation (a no-fight period of 2 weeks) causes a significant increase in their aggressiveness. This paper is aimed at studying—after a period of fighting deprivation—the involvement of genes (associated with neurotransmitter systems within the nucleus accumbens) in the above phenomena. The nucleus accumbens is known to participate in reward-related mechanisms of aggression. We found the following differentially expressed genes (DEGs), whose expression significantly differed from that in controls and/or mice with positive fighting experience in daily agonistic interactions followed by fighting deprivation: catecholaminergic genes *Th*, *Drd1*, *Drd2*, *Adra2c*, *Ppp1r1b*, and *Maoa*; serotonergic genes *Maoa*, *Htr1a*, *Htr1f*, and *Htr3a*; opioidergic genes *Oprk1*, *Pdyn*, and *Penk*; and glutamatergic genes *Grid1*, *Grik4*, *Grik5*, *Grin3a*, *Grm2*, *Grm5*, *Grm7*, and *Gad1*. The expression of DEGs encoding proteins of the GABAergic system in experienced aggressive male mice mostly returned to control levels after fighting deprivation, except for *Gabra5*. In light of the conceptual paradigm for analyzing data that was chosen in our study, the aforementioned DEGs associated with the behavioral pathology can be considered responsible for consequences of aggression followed by fighting deprivation, including mechanisms of an aggression relapse.

## 1. Introduction

Effects of repeated experience of one’s own aggression on behavior, physiology, and neurochemistry in animals have been studied purposefully by J.P. Scott [1,2], P.F. Brain with colleagues [3,4,5,6,7], and J.M. Koolhaas with colleagues [8,9].

Research on the influence of long positive fighting experience in daily agonistic interactions on mice has also been the focus of N.N. Kudryavtseva with colleagues for a long period (e.g., [10,11,12,13,14,15,16,17,18,19,20,21,22,23,24]). Experimental data indicate that male mice that had a repeated aggressive experience accompanied by wins develop a behavioral psychopathology [11,12,13,17,18], which has been verified by data on the development of psychoneurological symptoms. It has been shown that repeatedly aggressive and winning male mice after 20 days of agonistic interactions demonstrate changes in individual and social behaviors, such as enhanced impulsivity and aggressiveness, disturbances in social recognition, hyperactivity, stereotypic uncontrolled behaviors, and symptoms of autistic spectrum disorders. After a period of fighting deprivation (without agonistic interactions for 2 weeks), these male mice demonstrate increased aggression (in comparison with the period before deprivation), which is accompanied by preservation or enhancement of all types of pathological behavior seen in the aggressive animals [13,25,26]. It has been obvious that the repeated positive fighting experience in daily agonistic interactions with male mouse partners is accompanied by the development of a psychosis-like state with signs of an addiction-like state [12,13,25,26] or manic-like behavior [27] in mice. A neurochemical study on brain changes in aggressive male mice has revealed the activation of dopaminergic systems in the ventral tegmental area (VTA) and in the dorsal striatum (STR) and inhibition of serotonergic system activity in midbrain raphe nuclei (MRNs) [11,12,13].

It has been well-known that the main neurotransmitter systems—catecholaminergic (CAergic), serotonergic, opioidergic, GABAergic, and glutamatergic—are involved in the mechanisms of reward, drug abuse, addiction, and aggression (e.g., [6,12,28,29,30,31,32,33]). Our neurotranscriptomic and cellular studies on mice experienced in aggression have revealed alterations of the expression of numerous genes specific to brain regions depending on their main functions in the regulation of aggression mechanisms [16,18,19,20,21,22,23,24,34]. For example, in the VTA, which is the main brain region involved in reward mechanisms, an increase has been revealed in the expression of CAergic (*Th*, *Ddc*, *Slc6a2*, and *Slc6a3*), glutamatergic (*Slc17a7* and *Slc17a8*), and opioidergic (*Oprk1*) genes (and others) encoding relevant proteins [16]. Moreover, we have previously shown that in chronically winning (aggressive) mice, there is an increase in the proliferation of progenitor neurons and in the production of young neurons in the dentate gyrus (in the hippocampus), and these neurogenesis parameters remain modified during 2 weeks of fighting deprivation [15]. This means that hippocampal transcriptomic changes are associated with enhanced neurogenesis and take part in the formation of behavioral features in mice that have had a positive fighting experience in agonistic interactions as well as during subsequent fighting deprivation. Some of these genes in the hippocampus have been associated with behavioral traits, including abnormal aggression-related behavior, an abnormal anxiety-related response, and other traits [24]. The transcriptomic changes of gene expression are reversed only partially after a period of no fights. Similar data have been obtained regarding the STR [21].

We believe that genes encoding proteins responsible for the aggravation of the pathological condition in aggressive mice after 2 weeks of deprivation from fighting (aggression relapse) retain altered expression (compared to the control level) after the no-fight period. This supposition is based on our observations of alterations of social and individual behaviors and of the emotional state (reviews: [12,13,17,25]) that persist in aggressive mice for at least 2 weeks after the removal of the psychopathogenic external factor, that is, after a period without agonistic interactions. This persistence can serve as an indicator of the development of a psychoneurological disorder. Here, we are extending this view to other psychopathologies that may arise in mice in the course of a similar experiment. Our conceptual paradigm of data analysis is based on the notion that disease manifestation can result from the participation of pathology-related genes that retain altered expression for a long time, even after cessation of the exposure to a pathological stimulus.

There has been interest in the role of the mesolimbic dopaminergic circuit in the control over aggression reward (review [35,36,37]) because dopaminergic projections from the VTA to the nucleus accumbens (NAcc) modulate both aggression intensity and dopamine (DA) levels [38,39]. Our attention in this research has been concentrated on neurotransmitter genes in the NAcc, which is involved in reward mechanisms [36,40,41] and consequences of the repeated experience of aggression in mice [16,19,21,24].

The present study is focused on the differentially expressed genes (DEGs) associated with CAergic, serotonergic, opioidergic, glutamatergic, and GABAergic systems’ activities in the NAcc of mice having had repeated positive aggressive experience for 20 days and those after 2 weeks of deprivation from fighting. To obtain these data, transcriptomic analysis was performed. The study was carried out within the research field that we are developing, “Functional neurotranscriptomics of pathological conditions,” by means of the sensory contact model [10], which was later renamed the “chronic social conflict model.”

In this experiment, three groups of animals were analyzed: (1) controls (C), i.e., mice without a consecutive experience of agonistic interactions; (2) A20 winners, i.e., a group of mice aggressive daily for 20 days; and (3) AD (aggression deprivation), i.e., fight-deprived winners that were repeatedly aggressive for 20 days and then subjected to 14 days of deprivation (the no-fight period, without agonistic interactions). A graphical outline of the experiment is given in the Materials and Methods (Section 4).

## 2. Results

### 2.1. The Winners’ Behavior During Agonistic Interactions

**Figure 1:**
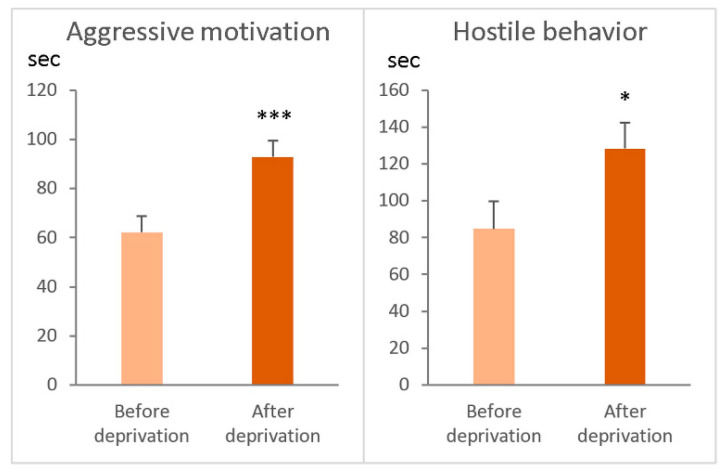
Total duration of aggressive motivation and hostile behavior demonstrated by experienced aggressive mice before (A20 mice, *n* = 10) and after fighting deprivation (AD mice, *n* = 10). * *p* < 0.05; *** *p* < 0.001, Wilcoxon test.

Among the winners with a positive fighting experience in 20 tests (for 20 days), the most aggressive animals were identified, which demonstrated in daily agonistic interactions the longest time of aggressive motivation (in seconds) as a behavioral reaction to a partner in the neighboring compartment of the cage before agonistic interaction in the partition test and hostile behaviors in the agonistic interaction test including the amount of time (s): total duration of attacks and duration (s) of aggressive grooming and diggings Figure 1).

Using the timetable of the experiment, it was shown (Figure 1) that aggressive motivation (total time spent near the partition as a reaction to the partner in the neighboring compartment of the cage) and total duration of hostile behavior during the agonistic test were significantly greater in AD mice than in A20 mice. This means that experimental groups of mice demonstrated the main symptom of the behavioral pathology, an increase in aggressiveness after the period of deprivation, as well as enhanced hyperactivity and aggressive motivation toward unaggressive partners.

### 2.2. Detecting DEGs in Pairwise Comparisons

Three-way comparisons of gene expression data between control, A20, and AD groups were performed according to ref. [42]. This analysis yielded 2629 DEGs for the “control versus A20” comparison, 1111 DEGs for the “control vs. AD” comparison (Table 1), and 2578 DEGs for the “A20 vs. AD” comparison (*p* < 0.05). This finding indicated that in NAcc of AD mice, the number of DEGs is decreased, tending to return to the control level.

### 2.3. DEGs of Neurotransmitter Systems

#### 2.3.1. CAergic Genes

**Figure 2:**
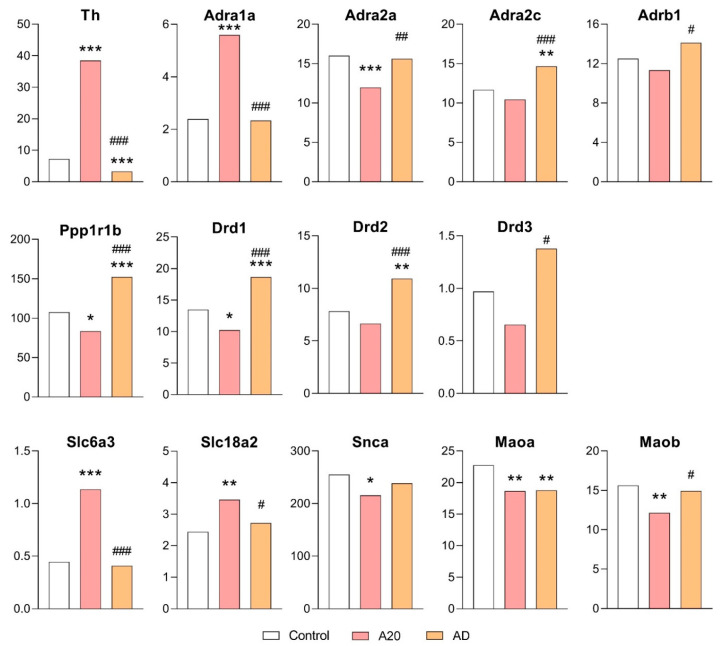
CAergic-system-associated DEGs in the NAcc. Data are presented in FPKM units. C: male mice with no consecutive experience of agonistic interactions; A20: aggressive male mice with consecutive 20 days of wins in daily agonistic interactions; AD: A20 males after a period of fighting deprivation for 14 days. C vs. A20 and C vs. AD—* *p* < 0.05; ** *p* < 0.01; *** *p* < 0.001; A20 vs. AD—^#^ *p* < 0.05; ^##^ *p* < 0.01; ^###^ *p* < 0.001. Additional statistical information is shown in Supplementary Table S1.

It was found (Figure 2, Supplementary Table S1) that 4 out of 14 DEGs in the C vs. A20 comparison were upregulated: *Th*, *Slc6a3*, *Adra1a*, and *Slc18a2*. Genes *Adra2a*, *Drd1*, *Maoa*, *Maob*, *Ppp1r1b*, and *Snca* were downregulated. After the period of aggression deprivation (C vs. AD comparison), a return to the control level of expression was registered for genes *Slc6a3*, *Adra1a*, *Adra2a*, *Maob*, *Snca*, and *Slc18a2*. In this comparison, there was increased expression of genes *Adra2c*, *Drd1*, *Drd2*, and *Ppp1r1b* and decreased expression of the *Th* gene in AD mice relative to the control and A20 mice. The expression of *Drd3* and *Adrb1* in AD mice did not differ from that in the control but was higher than that in A20 mice.

In light of our conceptual paradigm of data analysis, these results of the comparison of C vs. AD mice can be interpreted as follows. *Adra2c* (the gene encoding adrenoreceptors 2c), *Drd1* and *Drd2* (genes encoding DA receptors), *Th* (the gene encoding the rate-limiting enzyme of the catecholamine synthesis), and *Maoa* (the gene coding for mitochondrial enzymes that catalyze oxidative deamination of all monoamines) can be considered genes associated with the pathology. The *Ppp1r1b* gene (encoding the DARPP-32 protein) mediates the actions of DA and regulates the stimulation of dopaminergic and glutamatergic receptors. The *Ppp1r1b* shows the highest expression in AD mice in the absence of DA after aggression deprivation (Figure 2). It is well-known that DARPP-32 is crucial for the regulation of transcriptional and behavioral responses to pharmacological agents, including neuroleptics, antidepressants, and drugs of abuse, and it may serve as a therapeutic target in neurological and psychiatric disorders (review, [43]).

We assume that these genes associated with CAergic systems in the NAcc can be responsible for the development of reward-related effects of wins in the agonistic interactions, and these effects are the main symptoms of the pathological state similar to a psychosis-like disorder.

The present neurotranscriptomic analysis confirmed our previous finding that repeated aggression is accompanied by an increase in the activity of brain dopaminergic systems [44], as evidenced by elevated DOPAC (3,4-dihydroxyphenylacetic acid) levels and/or increased DOPAC/DA ratios in the olfactory bulbs, amygdala, hippocampus, NAcc, STR, and midbrain in the winners. Other authors have reported elevation of DA levels in the NAcc before, during, and after fights in aggressive rats [28,38]. In addition to these data, in the VTA (containing somata of mesolimbic dopaminergic neurons and playing an important role in the mediation of reward processes), overexpression of genes *Th*, *Dat1 (Slc6a3)*, and *Snca* has been found in aggressive winners [34,45]. Elevated mRNA levels of *Th* and *Slc6a3* persisted in the winners after the deprivation period in our work. The persistence of altered dopaminergic gene expression confirmed the development of a psychopathology in the winners. Of note, the level of aggressiveness—measured as latency to the first attack, the number of attacks, and total time spent attacking—is reported to positively correlate with *Th* and *Snca* mRNA levels in the VTA [34,45]. Thus, activation of dopaminergic systems during repeated positive fighting experience enhances the expression of dopaminergic genes in the VTA and, according to our present experiments, in the NAcc, which are the main reward-mediating brain regions.

In our previous study, we used an experimental approach to investigate a psychotropic drug’s effects under simulated clinical conditions [46]. The pharmacological data confirmed alterations of dopaminergic activity in the brain of repeatedly aggressive mice. Haloperidol, an antagonist of dopaminergic receptors, effectively inhibits aggression in male mice with a short (2-day) experience of aggression but is ineffective in the winners with 20-day experience [47]. Dopamine D1 receptor antagonist SCH-23,390 has a similar effect [48]. This means that dopaminergic receptors may be sensitized or desensitized to the blocking effects of DA receptor antagonists depending on the duration of the aggressive experience.

#### 2.3.2. Opioidergic and Cannabinoidergic Genes

**Figure 3:**
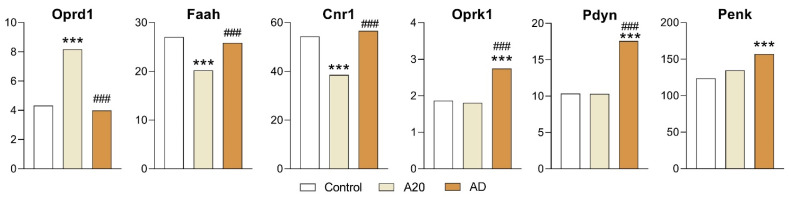
Opioidergic-system-associated and cannabinoidergic-system-associated DEGs in the NAcc. Data are presented in FPKM units. C: male mice without experience of agonistic interactions; A20: aggressive males with 20 consecutive days of wins in daily agonistic interactions; AD: A20 mice deprived of fighting for 14 days. C vs. A20 or C vs. AD—*** *p* < 0.001; A20 vs. AD—^###^ *p* < 0.001. Additional statistical information is shown in Supplementary Table S1.

Increased expression of *Oprd1* gene (encoding opioidergic 1d receptors) and decreased expression of *Cnr1* (encoding cannabinoid receptor 1, associated with cannabis dependence and abuse) and *Faah* (fatty acid amide hydrolase, which catalyzes the hydrolysis of the endocannabinoid 2-arachidonoylglycerol) were found in comparison with the control (C vs. A20 comparison; Figure 3; Supplementary Table S1). Nonetheless, their expression returned to the control level after fighting deprivation (in AD mice).

In the C vs. A20 comparison, there was no significant difference in expression for the following genes: *Oprk1* (encoding kappa opioid receptors) and *Pdyn* (whose product is proteolytically processed to form secreted opioid peptides beta-neoendorphin, dynorphin, leu-enkephalin, rimorphin, and leumorphin; these peptides are ligands for the kappa-type of opioid receptor). Dynorphin is involved in the modulation of responses to several psychoactive substances, including cocaine. A similar absence of differences in expression was observed for the *Penk* (proenkephalin) gene, which produces pentapeptide opioids Met-enkephalin and Leu-enkephalin, which are stored in synaptic vesicles and then released into the synapse, where they bind to mu- and delta-opioid receptors.

In contrast, after the period of fighting deprivation, the expression of *Oprk1* and *Pdyn* increased significantly in the comparison of A20 vs. AD mice, and *Penk* expression increased significantly in the C vs. AD comparison (Figure 3). Thus, these three genes may participate in the consequences of deprivation and may be involved in the manifestation of an aggression relapse. Additionally, in our previous study, we detected upregulation of opioidergic genes in some brain regions, including the *Oprk1* gene in the VTA and *Oprd1* and *Penk* in the prefrontal cortex of A20 mice compared with the control [16].

On the basis of previous pharmacological experiments, we have hypothesized that chronic activation of the brain’s opioid systems in aggressive winners leads to opioid drug tolerance similar to that of human addicts. Various experiments have shown that naltrexone (an opioid receptor antagonist) effectively reduces aggressiveness in male mice with a short experience of aggression, and, at the same doses, it is ineffective in male mice with a long history of aggression [49,50,51]. Selective mu-opioid receptor agonist morphine has a stimulatory effect on the locomotor activity of control mice and no effect on locomotor activity in 60% of the winners after fighting deprivation [25]. Desensitization of kappa-opioid receptors to selective agonist U50,488 in experienced aggressive winners has been documented, too [52,53]. This effect is associated with underexpression of the gene coding for kappa-opioid receptors in the VTA of male mice after 10 days of agonistic interactions [54].

Thus, opioidergic receptors could be sensitized or desensitized depending on the amount of aggressive experience and the severity of aggressiveness in mice. In this context, recurrent aggression and its enhancement after fighting deprivation has been explained by a deprivation (withdrawal) effect due to the involvement of opioidergic systems in the reward-related effects of repeated aggression. Possibly, similarly to humans, these findings indicate the development of tolerance to endogenous opioids (for example, the brain’s endogenous morphine) [55,56,57] or an opioid deficiency in the absence of DA inducing positive reinforcement.

It is well-known that the opioidergic and cannabinoidergic genes are involved in the opioid-mediated mechanisms underlying social interaction, attachment, and aggression [6,58] and in the formation of addiction states during repeated activation by drugs with rewarding effects (inducing good mood and positive emotions in humans). Our current experiments revealed that the acquisition of a positive fighting experience in daily agonistic interactions is accompanied by positive emotions induced by the wins; aggressive males every day demonstrate strong aggressive motivation toward the defeated male mice (losers) and repeatedly attack them even in the absence of the need to demonstrate superiority.

#### 2.3.3. Serotonergic Genes

**Figure 4:**
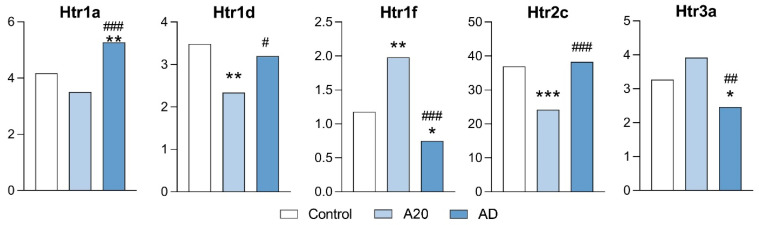
Serotonergic-system-associated DEGs in the NAcc. Data are presented in FPKM units. C: male mice with no experience of agonistic interactions; A20: males with 20 consecutive days of wins in daily agonistic interactions; AD: males with 20 consecutive days of wins next deprived of fighting for 14 days. C vs. A20 or C vs. AD—* *p* < 0.05; ** *p* < 0.01; *** *p* < 0.001; A20 vs. AD—^#^ *p* < 0.05; ^##^ *p* < 0.01; ^###^ *p* < 0.001. Additional statistical information is given in Supplementary Table S1.

In this experiment, in six out of the eight genes encoding proteins participating in regulation of serotonergic system activity (*Htr1d*, *Htr1f*, *Htr2c*, *Maoa*, *Maob*, and *Slc18a2*), expression changed in the C vs. A20 comparison (Figure 2 and Figure 4), with upregulation of *Slc18a2* and *Htr1f* and downregulation of *Htr2c*, *Htr1d*, *Maoa*, and *Maob* expression. After the period of aggression deprivation (C vs. AD), a return to the control level of expression of *Htr2c*, *Htr1d*, *Maob*, and *Slc18a2* was noted. In this comparison, increased expression of the *Htr1a* gene and decreased expression of *Maoa* were registered after deprivation compared with the control. Decreased expression of genes *Maoa*, *Htr1f*, and *Htr3a* and increased expression of *Htr1a*, *Htr1d*, *Htr2c*, and *Maob* were detected in the A20 vs. AD comparison (Figure 2 and Figure 4). We suppose that downregulation of *Htr1f*, *Htr3a*, and *Maoa* and upregulation of *Htr1a* in C vs. A20 and C vs. AD comparisons may be a specific effect of fighting deprivation, and these genes may be regarded as participating in the development of the pathological state of the AD mice.

A reduced 5-hydroxyindoleacetic acid (5-HIAA) level and/or 5-HIAA/5-HT ratio [11,12,44] and a decrease in the activity of a rate-limiting enzyme of serotonin (5-HT) biosynthesis (i.e., tryptophan hydroxylase) in the midbrain and STR have been demonstrated earlier in male mice with repeated aggressive experience [59,60]. Later, a transcriptomic analysis revealed alterations of the expression of serotonergic genes involved in synthesis, inactivation, and receptor sensitivity, as detected in different brain regions. For example, in aggressive mice with repeated positive fighting experience, genes *Tph2*, *Ddc*, *Slc6a4*, *Htr2a*, *Htr3a*, and *Htr5b* are downregulated in MRNs, containing mostly the somata of serotonergic neurons [14,61]. In the hypothalamus, genes *Maoa*, *Htr2a*, and *Htr2c* are downregulated, too. These data have confirmed the inhibition of serotonergic activity under the influence of repeated aggression [11]. In contrast, after the period of fighting deprivation, the expression of the *Tph2* and *Slc6a4* (Sert) genes is significantly higher compared to the period before deprivation, thereby returning to the normal (control) level, while the mRNA level of *Maoa* and *Htr1a* remains at a significantly higher level in MRNs compared with respective controls [14]. The change in serotonergic activity during the acquisition of aggressive experience has also been confirmed by pharmacological studies. Specific changes in the pharmacological sensitivity of 5-HT1A receptors to agonist buspirone have been documented [62]. All of these findings are consistent with the hypothesis of 5-HT deficiency in the brain of mice experienced in aggression [11,12].

The concept of a major inhibitory influence of 5-HT on aggressive behavior in animals and humans has been widely accepted by many investigators. Reduced cerebrospinal fluid levels of a major 5-HT metabolite called 5-HIAA, as well as decreased sensitivity of 5-HT1A receptors, have been shown in prisoners or psychiatric patients with a history of repeated violent or aggressive acts [63,64,65] and in rodents and nonhuman primates [66], thus possibly indicating a reduced inhibitory serotoninergic function in aggressive individuals.

We propose that the return of the expression of *Htr2c*, *Htr1d*, *Maob*, and *Slc18a2* to the control level after the period of fighting deprivation implies that changes in brain serotonergic activity in the NAcc are not the main cause of the behavioral pathology developing in the male mice in this experimental context. It has been suggested that reduced serotonergic activity stimulates the manifestation of aggression in conflict situations owing to the development of increased impulsivity [11,61]. On the contrary, here we show that changes in activity of the serotonergic system in the NAcc may be related to expression levels of the *Htr1a*, *Htr1f*, *Htr3a*, and *Maoa* genes. Moreover, the roles of different brain regions in the formation of impulsivity in individuals may differ.

In addition, our data support the idea that the repeated experience of aggression creates an imbalance between activities of two neurotransmitter systems—serotonergic and dopaminergic [12]—namely, inhibition and activation of these monoaminergic systems, respectively.

#### 2.3.4. GABAergic Genes

**Figure 5:**
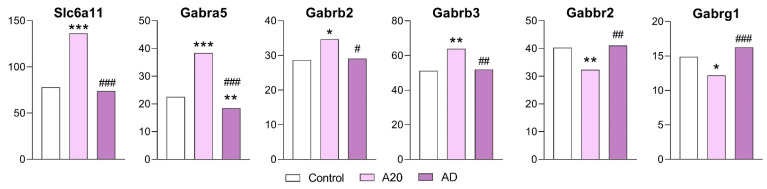
GABAergic-system-associated DEGs in the NAcc. Data are presented in FPKM units. C: mice with no experience of agonistic interactions; A20: male mice with 20 consecutive days of wins in daily agonistic interactions; AD: male mice with 20 consecutive days of wins in daily agonistic interactions next deprived of fighting for 14 days. C vs. A20 or C vs. AD—* *p* < 0.05; ** *p* < 0.01; *** *p* < 0.001; A20 vs. AD—^#^ *p* < 0.05; ^##^ *p* < 0.01; ^###^ *p* < 0.001. Additional statistical information is presented in Supplementary Table S1.

The expression of only 6 out of the 23 genes related to the functioning of the GABAergic system was found to change; the expression of *Slc6a11*, *Gabra5*, *Gabrb2*, and *Gabrb3* increased, and *Gabbr2* and *Gabrg1* expression decreased in the C vs. A20 comparison. After the period of deprivation, the expression of all genes, except for the *Gabra5* gene, returned to the control levels (Figure 5).

Therefore, the GABAergic system in the NAcc can be considered a system participating in the development of the behavioral pathology during repeated aggression, but it seems not to play a crucial role in the manifestation of the aggression relapse. Of note, earlier, we detected downregulation of GABAergic genes in the STR (*Gabra2*, *Gabra3*, *Gabrb2*, *Gabrg2*, and *Gabrg3*) and upregulation of some genes in the VTA (*Gabra1* and *Gabrg2*) of A20 mice [16]. Those data led us to suppose that the changes in gene expression may depend on the neurochemical environment, which differs among brain regions.

#### 2.3.5. The Glutamatergic System

Among 24 glutamatergic DEGs in A20 and/or AD mice, there were seven genes coding for metabotropic receptors (*Grm1*, *Grm2*, *Grm3*, *Grm4*, *Grm5*, *Grm7*, and *Grm8*), 12 genes encoding ionotropic receptors (*Gria3a Gria4*, *Grid1*, *Grid2ip*, *Grik1*, *Grik3*, *Grik4*, *Grik5*, *Grin1*, *Grin2a*, *Grin2d*, and *Grin3a*), two genes encoding glutamate decarboxylase (*Gad1* and *Gad2*), and two genes coding for inorganic transporters, *Slc17a7* and *Slc17a8* (Supplementary Table S1).

**Figure 6:**
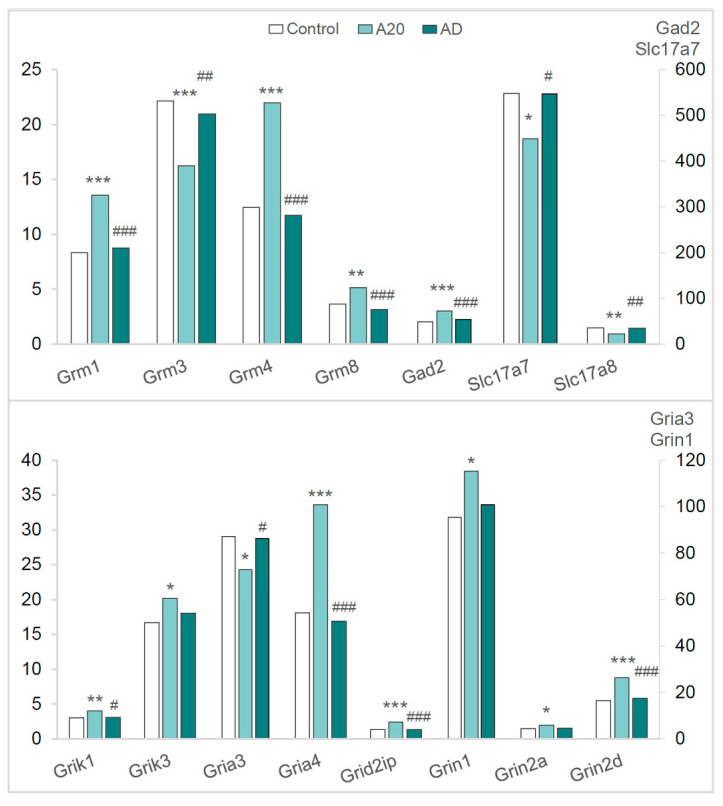
Glutamatergic-system-associated DEGs in the NAcc. Data are presented in FPKM units. C: male mice without consecutive experience of agonistic interactions; A20: male mice with 20 consecutive days of wins in daily agonistic interactions; AD: A20 male mice after the period of fighting deprivation for 14 days. C vs. A20 or C vs. AD—* *p* < 0.05; ** *p* < 0.01; *** *p* < 0.001; A20 vs. AD—^#^ *p* < 0.05; ^##^ *p* < 0.01; ^###^ *p* < 0.001. Additional statistical information is shown in Supplementary Table S1.

**Figure 7:**
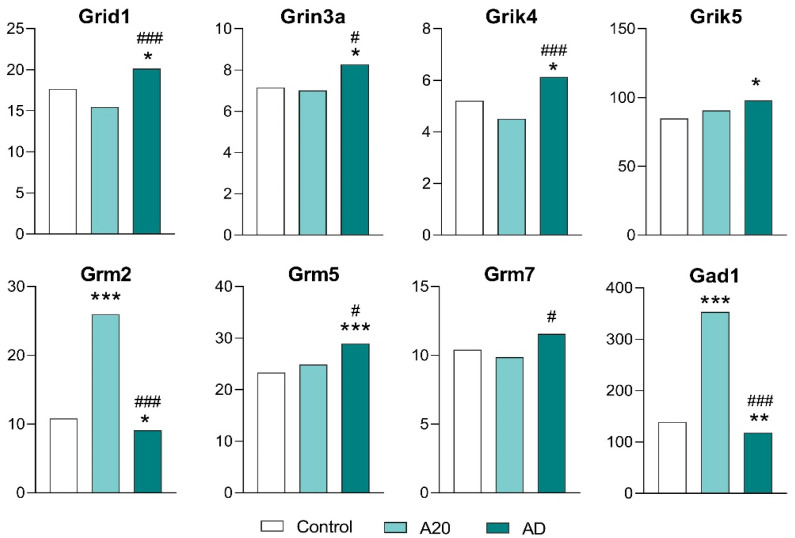
Glutamatergic-system-associated DEGs in the NAcc. Data are presented in FPKM units. C: male mice without consecutive experience of agonistic interactions; A20: male mice with 20 consecutive days of wins in daily agonistic interactions; AD: A20 male mice after the period of fighting deprivation for 14 days. C vs. A20 or C vs. AD—* *p* < 0.05; ** *p* < 0.01; *** *p* < 0.001; A20 vs. AD—^#^ *p* < 0.05; ^###^ *p* < 0.001. Additional statistical information is given in Supplementary Table S1.

In the comparison of C vs. A20 mice, 13 genes (*Grin2a*, *Grin2d*, *Grid2ip*, *Grik1*, *Grik3*, *Gria4*, *Grin1*, *Grm1*, *Grm2*, *Grm4*, *Grm8*, *Gad1*, and *Gad2*) proved to be upregulated. Four genes (*Gria3*, *Grm3*, *Slc17a7*, and *Slc17a8*) were found to be downregulated. For six genes (*Grid1*, *Grin3a*, *Grik4*, *Grik5*, *Grm5*, and *Grm7*), expression was unchanged (Figure 6, Figure 7, Supplementary Table S1).

In the comparison of C vs. AD mice, 15 genes (*Gad2*, *Gria3*, *Gria4*, *Grid2ip*, *Grik1 Grik3*, *Grin1*, *Grin2a*, *Grm1*, *Grm3*, *Grm4*, *Grm8*, *Grin2d*, *Slc17a7*, and *Slc17a8*) returned to the control expression level after aggression deprivation (Supplementary Table S1, Figure 6).

After the period of fighting deprivation in the C vs. AD comparison, genes *Grid1*, *Grin3a*, *Grik4*, *Grik5*, and *Grm5* were upregulated, and two genes (*Gad1* and *Grm2*) were downregulated. In the comparison of A20 vs. AD mice, the expression of *Grm7* differed (Figure 7, Supplementary Table S1).

According to our conceptual paradigm of data analysis, glutamatergic genes *Grid1, Grin3a, Grik4, Grik5, Grm2, Grm5*, and *Grm7* (encoding ionotropic and metabotropic receptors) and the *Gad1* gene (encoding glutamate decarboxylase) may be regarded as genes associated with the observed pathology (Figure 7). Expression levels of these genes differed in the C vs. AD comparison. Expression of *Grm7* gene differed in the A20 vs. AD comparison.

In summarizing the roles of both GABAergic and glutamatergic systems, all GABAergic DEGs (except for the *Gabra5a* gene), which encode GABA-A receptors (ligand-gated chloride channels), and 15 glutamatergic DEGs returned to the control expression level in AD mice after the deprivation period. Consequently, these genes cannot be regarded as participating in the development of the aggression-related pathology. In contrast, eight glutamatergic genes—*Grin3a*, *Grik4*, *Grid1*, *Grik5*, *Grm2*, *Grm5*, *Grm7* and *Gad1*—may be involved in the mechanism of relapse.

Moreover, when analyzing the role of GABAergic and glutamatergic systems, other authors [32] came to the conclusion that, for example, drug-addicted individuals can show widespread abnormalities in brain neurochemistry and function (including the systems of excitatory and inhibitory neurotransmitters glutamate and γ-aminobutyric acid-GABA, respectively, which are disturbed in addiction) that vary depending on individual characteristics (e.g., abstinence duration) and, as we propose, depending on brain regions (as demonstrated in our analyses of glutamatergic and/or GABAergic abnormalities).

### 2.4. DEGs Associated with the Observed Pathology

**Figure 8:**
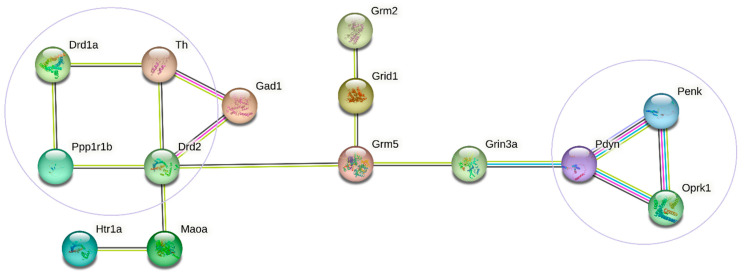
The functional enrichment network constructed by means of the STRING database (https://string-db.org/; accessed on 10 August 2023) from the DEGs associated with the activity of neurotransmitter systems in the NAcc. Lines are protein–protein associations. Purple lines indicate experimentally determined interactions; blue lines denote known interactions from curated databases; black lines show co-expression; lilac lines mean protein homology; and green lines represent the results of text mining.

Within the framework of the proposed paradigm, the genes significantly differing in expression compared with the level after fighting deprivation (C vs. AD and A20 vs. AD) can be considered genes implicated in pathologically aggressive behavior. According to the data discussed above, these include CAergic genes (*Th*, *Drd1*, *Drd2*, *Adra2c*, *Ppp1r1b*, and *Maoa*), serotonergic genes (*Htr1a*, *Htr3a*, *Htr1f*, and *Maoa*), opioidergic genes (*Oprk1*, *Pdyn*, and *Penk*), a GABAergic gene (Gabra5), and glutamatergic genes (Grin3a, *Grik4*, *Grid1*, *Grik5*, *Grm2*, *Grm5*, *Grm7*, and *Gad1*). Figure 8 presents the DEGs associated with the activity of neurotransmitter systems in the NAcc. This network was constructed by means of the STRING database. DEGs *Adra2c*, *Htr1f*, *Gabra5*, *Grik4*, *Grik5*, *Grm7*, and *Htr3a* were not included in relevant high-level interactions according to the STRING database.

The STRING database (https://string-db.org/) revealed two independent relationships between the DEGs associated with CAergic systems (*Drd1*, *Drd2*, *Th*, and *PPP1r1b*) and opioidergic genes (*Oprk1*, *Pdyn*, and *Penk*), which are responsible for different processes. Nevertheless, these two groups are interconnected through glutamatergic gene *Grm5* (encoding metabotropic receptors) and glutamatergic gene *Grin3a* (encoding ionotropic receptors). This analysis suggests that the DEGs in question may contribute to the pathological addiction-like state emerging during the positive fighting experience.

Additionally, we found significant correlations between expression levels of these pathology-associated genes in control mice, in A20 mice, and in AD mice (Supplementary Table S2). A lower number of correlations between genes was found in control mice (1–6 correlations for some genes, 74 total), suggesting that most of these genes are independent from one another in intact mice.

The largest number of correlations between expression levels of pathology-associated genes was noticed in A20 mice (Supplementary Table S2). There were 229 significant correlations between genes, including 15 correlations with *Grm5* and *Htr3a*, 13 with *Grik4*, *Grm5*, *Ppp1r1b*, and *Th*, 12 with *Adra2c*, 11 with *Grm7*, and 10 with *Drd1*, *Drd2*, *Drd3*, *Oprk1*, and *Pdyn*. This finding indicates high levels of transcriptional coordination within these gene clusters in A20 mice.

After the period of deprivation, we found significant correlations between pathology-related genes in AD mice and expression levels (0–9 correlations for some genes, 103 total). In all mouse groups, correlations between CAergic (*Adra2c*, *Drd1*, *Drd2*, *Drd3*, and *PPP1r1b*) and opioidergic (*Oprk1*, *Pdyn*, and *Penk*) genes were present (Table 2). It can be theorized that these coordinated changes in gene expression (unaffected by the positive fighting experience and aggression deprivation) are specific for the NAcc, which is responsible for reward-related mechanisms of aggression.

Of note, similar data have been obtained for human alcoholics [29]; the expression of DRD1 and DRD2 strongly correlates with that of PDYN and OPRK1, indicating dysregulation of the DYN/KOR system and of DA signaling because of (i) alterations in co-expression patterns of opioid genes and (ii) DRD1 underexpression. This observation allowed us to hypothesize similar mechanisms of formation of addictive states during an imbalance in the activity of D1-receptor-containing and D2-receptor-containing pathways if we compare human alcoholics with our winners with repeated experience of aggression.

## 3. Discussion

The aim of this study was to search for genes taking part in the development of a pathological condition similar to a psychosis-like state with signs of an addiction-like state during repeated positive fighting experience in daily agonistic interactions between mice, as shown earlier in our papers [12,13,25,26]. As a criterion for the selection of genes associated with the pathology, we chose the following conceptual paradigm of data analysis: after a 14-day period of fighting deprivation (without agonistic interactions and demonstration of aggression), these genes should differ significantly in their expression between AD mice and control mice and/or A20 mice without a return to baseline expression after the deprivation period.

In this work, we deal with the neurotransmitter genes whose products participate in CAergic, serotonergic, opioidergic, GABAergic, and glutamatergic regulatory systems in the NAcc, which, according to literature data, are involved in mechanisms of aggression accompanied by positive reinforcement and reward, as reported in experimental and psychological studies [35,36,37].

Summarizing the results of our study and examining our data within the proposed paradigm, a number of pathology-associated genes with changed expression in the NAcc can be considered, including the upregulated genes *Drd1*, *Drd2*, *Drd3*, *Adra2c*, *Ppp1r1b*, *Oprk1*, *Pdyn*, *Penk*, and *Htr1a* and the downregulated genes *Th*, *Maoa*, *Htr1f*, and *Htr3a.* The GABAergic *Gabra5* gene, as well as glutamatergic genes *Grin3a*, *Grik4*, *Grid1*, *Grik5*, *Grm2*, *Grm5*, *Grm7*, and *Gad1,* were also included according to our above-mentioned criteria. Most of these genes significantly correlated in expression changes between one another (Supplementary Table S2). We assume that dopaminergic genes in the NAcc are responsible for the formation of positive emotions and the development of a psychosis-like state during prolonged activation of CAergic processes. At the same time, opioidergic genes are involved in an addiction-like state, thereby promoting a relapse of aggressive behavior during withdrawal, accompanied by psychoneuropathological symptoms. According to the STRING database analysis, we can say that glutamatergic proteins encoded by genes *Grm5* (coding for metabotropic receptors) and *Grin3a* (encoding ionotropic receptors) can mediate the regulation of proteins encoded by the analyzed CAergic and opioidergic genes and the expression of these genes. The *Gad1* gene encoding glutamate decarboxylase is involved in the regulation of genes *Th* and *Drd2*.

We noticed a dynamic rearrangement of the functioning of genes during the formation of the observed behavioral pathology. In control animals, the number of correlations between pathology-associated genes is lower (79) than in the other groups of mice, which reflects the normal functioning of genes without any external perturbations. In A20 mice, with the development of the pronounced pathology of behavior, many genes seem to participate in its development (229 genes that correlate with one another in expression). After the period of fighting deprivation, it is possible to identify the genes that retain the altered expression (only 103 genes that correlate with each other).

Previously, it has been assumed on the basis of our studies [12,25,26] that during the repeated positive fighting experience, the natural innate mechanisms regulating aggressive behavior transform into pathological ones, which are based on neurochemical shifts in the brain. It has been hypothesized that under certain circumstances, effects of endogenous opioids in chronically aggressive individuals can be reversed and that the resultant emotional and physical discomfort will eventually lead to an internal drive to aggression or to an outbreak of aggression. Thus, with prolonged aggression, neurobiological mechanisms are activated that themselves stimulate aggression. This mechanism may be another reason for the aggression relapse. Now, we have demonstrated that dopaminergic and opioidergic genes encoding respective proteins in the NAcc play a major role in these processes.

We can also say that the psychosis-like state can be a consequence of high impulsivity developing during decreased serotonergic activity shown in many papers [12,14,61] and stimulated dopaminergic and opioidergic systems, accompanying withdrawal after the no-fight period in the winners. Nonetheless, one should keep in mind that the development of all of these states is determined by the duration of positive fighting experiences and, above all, by the social context and social environment that involve the absence of punishment and denunciation (in humans).

The same effects can be assumed in the development of the corresponding psychopathologies in humans, as escalated violence and aggression accompany the development of many psychiatric disorders, e.g., manic–depressive disorder, schizophrenia, compulsive–obsessive disorder, drug abuse, and autism (reviews: [67,68,69]). Recurrent aggressive behavior also takes place in professional sports, security services, and other occupations. The investigation of neurobiological mechanisms of (and factors provoking) repeated aggressive behavior is obviously an urgent task. Recent reviews on this subject [30,31,35,36,37,41,70] have attempted to shed light on this issue to confirm or refute previous notions about the rewarding effects of aggression attributed previously only to humans. Such animal studies have demonstrated that abnormal aggression can be excessive and serves as reinforcement, which can lead to a pathological state during repeated positive fighting experiences, including, in our case, a psychosis-like state with signs of an addiction-like state.

## 4. Materials and Methods

### 4.1. Animals

Adult C57BL/6 male mice were obtained from the Animal Breeding Facility, a branch of the Institute of Bioorganic Chemistry of the RAS (Pushchino, Moscow region). Animals were housed under standard conditions (at a constant temperature of 22 ± 2 °C under a 12:12 h light/dark regimen starting at 8:00 am, with food in pellets and water available ad libitum). Mice were weaned at 3 weeks of age and housed in groups of 8–10. The experimental cages were standard plastic cages. Experiments were performed on 10–12-week-old animals. All procedures were in compliance with European Communities Council Directive 210/63/EU of 22 September 2010. The protocol of the study was approved by Scientific Council No. 9 of the Institute of Cytology and Genetics SB RAS of 24 March 2010, decision No. 613 (Novosibirsk, http://spf.bionet.nsc.ru).

### 4.2. Implementation of the Repeated Aggressive Experience in Male Mice

**Figure 9:**
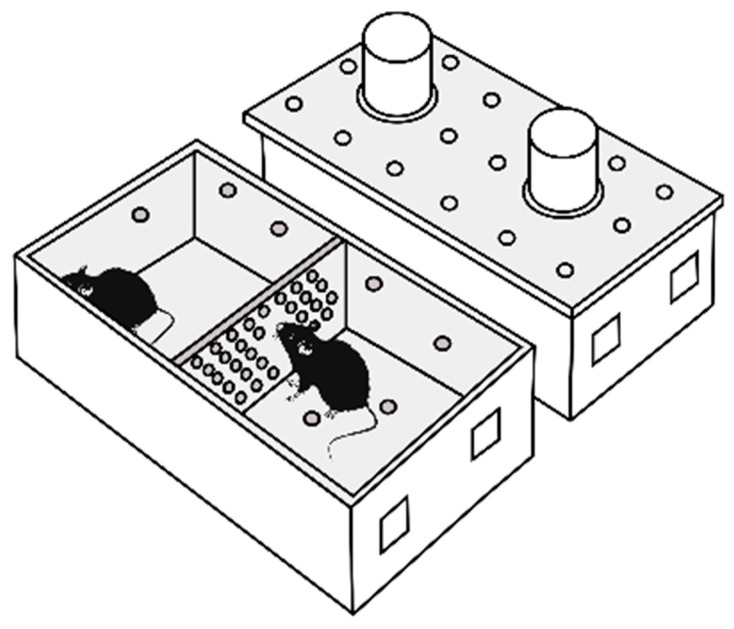
Experimental cage.

Repeated positive and negative social experiences in male mice were induced by daily agonistic interactions through the use of the sensory contact model [10], which was later renamed the “model of chronic social conflicts” and is described in detail in some reviews [13,17]. Pairs of male mice were each placed in a cage (28 × 14 × 10 cm) bisected by a transparent perforated partition, allowing the animals to see, hear, and smell each other but preventing physical contact [Figure 9].

**Figure 10:**
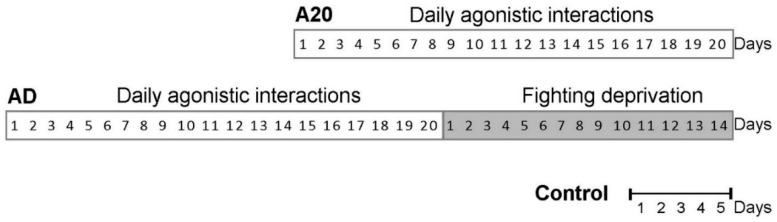
Timetable of the experiment. A20: the mice with 20-day repeated aggression accompanied by wins in the daily agonistic interactions; AD: A20 mice after 14 days of fighting deprivation; control: mice without consecutive agonistic interactions.

The animals were left undisturbed for 2 days to adapt to the new housing conditions and sensory contact before they were subjected to agonistic encounters. Every afternoon (2:00–5:00 p.m. local time), the cage cover was replaced by a transparent one, and 5 min later (the period necessary for activation), the partition was removed for 10 min to encourage agonistic interactions. The superiority of one of the mice was established within two or three encounters with the same opponent. The superior mouse would be chasing, biting, and attacking another, who would be demonstrating only defensive behavior (e.g., upright or sideways postures and withdrawal). To prevent damage to defeated mice, the aggressive interactions between males were discontinued by lowering the partition if the strong attacking behavior lasted for 3 min (in some cases, less). Each defeated mouse (loser) was exposed to the same winner for 3 days, whereas afterwards each loser was placed after the fight into an unfamiliar cage containing an unfamiliar winning partner behind the partition. Each aggressive mouse (winner) remained in its own cage. This procedure was performed once a day for 20 days and yielded equal numbers of losers and winners. In this study, intermittent aggression was chosen for the experiment, in contrast to the strong daily aggression that was used in our previous study [16] (we distinguish three types of winners: strongly aggressive, aggressive, and intermittently aggressive). After that, the winners were deprived of the agonistic interactions for 14 days, as the partition was not removed. The scheme of the experiment is presented in Figure 10.

### 4.3. Experimental Scheme

In this experiment, three groups of animals were analyzed: (1) controls (C), i.e., mice without a consecutive experience of agonistic interactions; (2) A20, winners, i.e., a group of mice daily intermittently aggressive during 20 days; and (3) AD, fight-deprived winners after 14 days of deprivation (no-fight period without agonistic interactions), before which they were repeatedly aggressive mice for 20 days.

### 4.4. Behavioral Tests for Aggressive Motivation and Hostile Behavior

Video recordings were carried out to describe the behavior of experimental animals in detail during the agonistic interactions. Among the winners (mice with positive fighting experience in 20 tests [for 20 days]), most highly aggressive animals were identified, which in daily agonistic interactions demonstrated the longest duration of aggressive motivation and hostile behaviors.

The partition test [71] was performed to estimate the aggressive motivation of mice toward the losers. The total time spent near the partition (moving near the partition, smelling and touching it with one or two paws, clutching and hanging, sticking the nose into the holes or gnawing the holes) were scored during 5 min as indices of reacting to the partner in the neighboring compartment of an experimental cage. The time during which the males showed a sideways posture or were “turning away” near the partition was not included in the total time scored. It has previously been shown that the time spent near the partition correlates with the severity of aggressive behavior, i.e., the duration of attacks during the test.

As parameters of hostile behaviors, we analyzed the following parameters during the test when the partition was removed: total duration of the attacks, biting, and chasing a partner in agonistic interactions during 10 min; total duration of digging (digging up and scattering the sawdust on the loser’s territory (kick digging or push digging the sawdust forward or backward using the forepaws or hind paws); and the total duration of aggressive grooming (the winner mounts the loser’s back, holds it down, and spends much time licking and nibbling at the loser’s scruff of the neck). It is regarded as a ritual form of aggression that effectively suppresses the other male mouse without physical effort, thereby replacing attacks. All of these activities aimed at inflicting physical or psychological damage on the conspecific.

The number of mice used for transcriptomic analysis was 6 for each animal group. The winners (A20) 24 h after the last agonistic interaction, fight-deprived winners (AD), and the control animals (C) were decapitated simultaneously. The NAcc was dissected by the same experimenter according to the map presented in the Allen Mouse Brain Atlas, 2021 [72]. All tissue samples were placed in an RNAlater solution (Life Technologies, Waltham, MA, USA) and stored at −70 °C until sequencing.

### 4.5. The NAcc in the Control of Aggressive Behavior

The NAcc is a part of the reward system and plays an important role in the processing of rewarding and reinforcing stimuli (addictive drugs, sex, and exercise). According to the literature (reviews, [73,74]), the NAcc is a principal target of dopaminergic neurons of the VTA, and it is considered a critical brain region involved in reward and drug dependence processes [75]. Most neurons in the NAcc are GABAergic medium spiny neurons (MSNs). The major projection neurons in this brain region are DA receptor DRD1- and DRD2-expressing neurons. Approximately 1–2% are cholinergic interneurons, and another 1–2% are GABAergic interneurons. GABAergic MSNs play an important part in the processing of reward stimuli [76], and they are regulated by DA from the VTA. Functional and molecular alterations occur within the NAcc in numerous psychoneurological pathologies, such as depression, addiction, schizophrenia, Huntington’s, Parkinson’s, and Alzheimer’s diseases, and alcoholism ([74], review).

Recent research interest is also concentrated on the role of the mesolimbic dopaminergic circuit in the control of aggression reward [35,36,37]. It has been reported that Drd1-expressing neurons control aggression self-administration and aggression seeking in mice [70,77]. Dopaminergic projections from the VTA to the NAcc modulate DA levels [38] and aggression intensity [78,79].

### 4.6. Statistical Analysis

Statistical analysis of the transcriptomic data was conducted using XLStat software (Version 2016.02, Addinsoft Inc., Paris, France) (www.xlstat.com, accessed on 31 March 2016). Aggressive motivation (total time spent near the partition as a reaction to the partner in the neighboring compartment of the cage) and total duration of hostile behavior during the agonistic test before and after fighting deprivation were compared using the Wilcoxon test. Pearson correlation analysis was used to evaluate the relationship between expression levels of pathology-associated genes.

### 4.7. RNA-Seq Data Analysis and Processing

The collected brain samples were delivered to Genoanalytica Ltd. (www.genoanalytica.ru, accessed on 18 December 2021, Moscow, Russia) for RNA-Seq sequencing. By means of the Dynabeads mRNA Purification Kit (Ambion, Thermo Fisher Scientific, Waltham, MA, USA), mRNA was extracted. Using the NEBNext mRNA Library PrepReagent Set for Illumina (New England Biolabs, Ipswich, MA, USA) according to the manufacturer’s protocol, cDNA libraries were created next. The target coverage was set to 20 million reads per sample on average. Cufflinks software (v2.2.1) was used to estimate gene expression levels in FPKM units (fragments per kilobase of transcript per million mapped reads) and to subsequently identify DEGs in the NAcc of male mice. Statistical analysis of the transcriptomic data was conducted using XLStat software (www.xlstat.com, accessed on 31 March 2016). C vs. A20, C vs. AD, and A20 vs. AD comparisons of gene expression data were performed. The differential transcription level is described using the criteria *p* value < 0.05 and q value < 0.05 (Supplementary Table S1).

For functional annotation of DEGs, the KEGG database was used to reveal the neurotransmitter genes [80], and the functional enrichment network was constructed by means of the STRING database [81].

### 4.8. The Genes That Were Analyzed in the NAcc Before and After the Deprivation Period

(Supplementary Table S3)

Catecholaminergic (CAergic) systems: *Th*, *Ddc*, *Dbh*, *Maoa*, *Maob*, *Comt*, *Slc6a2*, *Slc6a3*, *Slc18a2*, *Snca*, *Sncb*, *Sncg*, *Ppp1r1b*, *Drd1*, *Drd2*, *Drd3*, *Drd4*, *Drd5*, *Adra1a*, *Adra1b*, *Adra1d*, *Adra2a*, *Adra2b*, *Adra2c*, *Adrb1*, *Adrb2*, *Adrb3*, *Adrbk1*, and *Adrbk2*;

Opioidergic and cannabinoidergic systems: *Pdyn*, *Penk*, *Pomc*, *Pnoc*, *Oprm1*, *Oprd1*, *Oprk1*, *Opcml*, *Ogfr*, *Ogfrl1*, *Cnr1*, *Cnr2*, and *Faah*;

Serotonergic system: *Tph2*, *Ddc*, *Maoa*, *Maob*, *Htr1a*, *Htr1b*, *Htr2a*, *Htr2c*, *Htr3a*, *Htr4*, *Htr5b*, *Htr6*, *Htr7*, *Htr1d*, *Htr1f*, *Htr2b*, *Htr3b*, *Htr5a*, *Slc6a4*, and *Slc18a2*;

GABAergic system: *Gabra1*, *Gabra2*, *Gabra3*, *Gabra4*, *Gabra5*, *Gabra6*, *Gabrb1*, *Gabrb2*, *Gabrb3*, *Gabrg1*, *Gabrg2*, *Gabrg3*, *Gabrd*, *Gabre*, *Gabrp*, *Gabrq*, *Gabbr1*, *Gabbr2*, *Gabrr1*, *Gabrr2*, *Gabrr3*, *Slc6a11,* and *Slc6a13*;

Glutamatergic system: *Gria1*, *Gria2*, *Gria3*, *Gria4*, *Grik1*, *Grik2*, *Grik3*, *Grik4*, *Grik5*, *Grin1*, *Grin2a*, *Grin2b*, *Grin2c*, *Grin2d*, *Grin3a*, *Grin3b*, *Grm1*, *Grm2*, *Grm3*, *Grm4*, *Grm5*, *Grm6*, *Grm7*, *Grm8*, *Grid1*, *Grid2*, *Grid2ip*, *Gad1*, *Gad2*, *Slc17a6*, *Slc17a7*, and *Slc17a8*.

## 5. Conclusions

Previously, it was demonstrated that the accumulation of effects of positive fighting experience day to day is accompanied by significant changes in social and individual behaviors, multiple long-term changes in neurotransmitters’ synthesis and catabolism and receptors’ sensitivity, and in the expression of neurotransmitter-related genes in the brain, consistent with the development of a psychosis-like state with signs of an addiction-like state in mice [12,13]. After a no-fight period (aggression deprivation), the changes in the expression of numerous genes and enhanced aggressiveness persist for a long period, and this phenomenon is one of the reasons for the recurrent aggression (relapse) demonstrated in our experiments [13,25,26]. Now, we have pieces of evidence that numerous genes associated with major neurotransmitter systems (especially the opioidergic and CAergic systems) in the reward-related brain regions, particularly in the NAcc of mice, retain their altered expression after 2 weeks of a no-fight period (without agonistic interactions). In light of our proposed conceptual paradigm of data analysis, the revealed DEGs can be considered genes associated with the observed pathology that play a major role in the consequences of the repeated positive fighting experience.

## Figures and Tables

**Table 1:** Three-way comparisons based on detection of DEGs.

Comparisons	Number of DEGs	
	*p* Value < 0.05	q Value < 0.05
Control vs. A20	2629	1200
Control vs. AD	1111	232
A20 vs. AD	2578	1338

**Table 2:** Coefficients of positive correlations between expression levels of CAergic and opioidergic genes in the NAcc of AD male mice.

Variables	Adra2c	Drd1	Drd2	Drd3	Oprk1	Pdyn	Penk	Ppp1r1b
Adra2c	1	0.965	0.935	0.851	0.966	0.958	0.945	0.974
Drd1	0.965	1	0.983	0.947	0.991	0.990	0.983	0.993
Drd2	0.935	0.983	1	0.975	0.990	0.993	0.995	0.987
Drd3	0.851	0.947	0.975	1	0.944	0.948	0.959	0.945
Oprk1	0.966	0.991	0.990	0.944	1	0.996	0.997	0.997
Pdyn	0.958	0.990	0.993	0.948	0.996	1	0.995	0.990
Penk	0.945	0.983	0.995	0.959	0.997	0.995	1	0.991
Ppp1r1b	0.974	0.993	0.987	0.945	0.997	0.990	0.991	1

## Data Availability

All relevant data are available in the Supplementary Materials and from the authors.

## Supplementary Materials

The following supporting information can be downloaded at https://www.mdpi.com/article/10.3390/ijms26178580/s1, Supplementary Table S1: FPKM values for neurotransmitter DEGs in the NAcc; Table S2: Correlations between expression of genes and pathology; Table S3: Neurotransmitter genes analyzed in the NAcc before and after the deprivation period.

## Abbreviations

C	control
A20	groups of intermittently daily aggressive mice during 20 days
AD	A20 mice after 14 days of deprivation (no-fight period without agonistic interactions)
5-HT	serotonin
5-HIAA	5-hydroxyindolacetic acid
DA	dopamine
DOPAC	3,4-dihydroxyphenyleacetic acid
DEGs	differentially expressed genes
FPKM	fragments per kilobase of transcript per million mapped reads
CAergic	catecholaminergic
GABA	γ-aminobutyric acid
MRN	midbrain raphe nuclei
MSNs	medium spiny neurons
NAcc	nucleus accumbens
STR	dorsal striatum
VTA	ventral tegmental area
